# Prediction of trauma-induced coagulopathy in early polytrauma: a prospective cohort study

**DOI:** 10.3389/fcvm.2026.1819390

**Published:** 2026-06-15

**Authors:** Bo Li, Lichun Liu, Xiaoli Feng, Bohui Lv, Xiaojun Yang

**Affiliations:** 1 General Hospital, First Clinical Medical College, Ningxia Medical University, Yinchuan, China; 2 Emergency Department, Ningdong Hospital, Ningxia Hui Autonomous Region, Yinchuan, China

**Keywords:** logistic regression, polytrauma, predictive model, risk factors, traumatic induced coagulopathy

## Abstract

**Background:**

Traumatic induced coagulopathy (TIC) is a key factor affecting the prognosis of patients with polytrauma. Early identification of patients at high risk for TIC and timely initiation of damage-control interventions are crucial. This study aims to develop and validate a predictive model for individualized estimation of TIC risk based on clinical characteristics and routine laboratory indicators.

**Methods:**

This prospective cohort study included 183 adult patients, aged 18 to 85 years, with confirmed multiple injuries who presented to the emergency department of our hospital between July 1, 2023, and December 31, 2024. Patients were divided into a TIC group (*n* = 123) and a non-TIC group (*n* = 60) according to the occurrence of TIC. Candidate predictors were screened using univariate analysis, least absolute shrinkage and selection operator (LASSO) regression, and multivariable logistic regression to construct the model. Model performance was comprehensively evaluated using the area under the receiver operating characteristic curve (AUC), calibration curves, and decision curve analysis (DCA).

**Results:**

Four core predictors were ultimately identified: emergency surgery, pH value, red blood cell count (RBC), and Injury Severity Score (ISS).The model demonstrated good discriminative ability (AUC = 0.903, 95% CI: 0.858–0.948).The optimal cut-off value determined by the Youden index was 0.651, with a sensitivity of 81.3% and a specificity of 86.7%.The calibration curve showed high agreement between predicted and observed risks. Decision curve analysis further indicated that the model provided favorable clinical net benefit across a wide range of threshold probabilities.

**Conclusion:**

The predictive model developed in this study showed promising ability to estimate individualized TIC risk in patients with polytrauma and may serve as an effective tool to optimize the allocation of medical resources and facilitate early diagnosis and intervention for traumatic coagulopathy.

## 1 Introduction

Trauma is a widespread public health problem across countries worldwide. It remains one of the major global health challenges and a leading contributor to the global burden of disease, with both incidence and mortality continuing to rise (1). Life-threatening injuries in at least two anatomical regions (e.g., head, chest, abdomen) or systems (e.g., skeletal, nervous) at the same time (2). Owing to its clinical complexity, patients with severe polytrauma often present with homeostatic derangements and insufficient effective circulating blood volume, which may lead to endotoxin translocation, increased anaerobic metabolism, and inadequate tissue and organ perfusion. Consequently, polytrauma is associated with high rates of disability and mortality and represents a particularly severe type of injury (3, 4).

In China, it was estimated that 77.10 million new trauma cases occurred in 2017, including approximately 0.73 million deaths attributable to polytrauma, accounting for 7.00% of all deaths nationwide (5). Polytrauma has become the fifth leading cause of death, following malignancies, cerebrovascular disease, respiratory disease, and heart disease, and is also a major cause of disability and death among adults younger than 45 years (6, 7). Therefore, early intervention and timely rescue are of critical importance in patients with polytrauma.

Previous studies have shown that polytrauma is frequently accompanied by extensive tissue injury and massive hemorrhage (8), which can impair endothelial function, accelerate consumption of coagulation factors, and activate the fibrinolytic system, thereby disrupting the balance among anticoagulation, coagulation, and fibrinolysis. Together with trauma-related physiological dysregulation, stress responses, and hypoxia, these changes predispose patients to coagulopathy (9, 10). Traumatic induced coagulopathy (TIC) is a clinical syndrome characterized by an imbalance between anticoagulation and fibrinolysis. Evidence indicates that patients who develop TIC have substantially worse outcomes than those without TIC, with higher rates of organ dysfunction and sepsis and a significantly increased risk of death (11). Approximately 30% of severely injured patients already exhibit TIC upon arrival at the emergency department, resulting in greater transfusion requirements, increased use of hospital resources such as mechanical ventilation, longer ICU and hospital stays, and more challenging clinical management (12). Therefore, the aim of this study was to identify the risk factors for coagulation dysfunction in polytrauma patients and to evaluate the clinical value of early or pre-TIC (trauma-induced coagulopathy) active intervention strategies in improving patient outcomes and reducing mortality.

## 2 Materials and methods

### 2.1 Study population and data source

This study was a single-center prospective cohort study. We enrolled 183 patients with confirmed polytrauma who presented to the Emergency Department of Ningxia Medical University General Hospital between July 1, 2023, and December 31, 2024. During the study period, we consecutively screened multiple-trauma patients who presented to the emergency department and met the inclusion criteria.Relevant variables were collected using a standardized case report form. This was an observational study; written informed consent was obtained from each patient or their legal representative before enrollment. The study adhered to the principle of non-maleficence and protected patient privacy. Ethical approval was granted by the Medical Ethics Committee of Ningxia Medical University (Approval No.: KYLL-2023-0159; Yinchuan, China). All procedures complied with the Declaration of Helsinki.

#### 2.1.1 Diagnostic criteria

1. Polytrauma was defined as: (i) injuries caused by a single mechanical mechanism; (ii) simultaneous injuries involving two or more anatomical regions or organs; (iii) at least one injury that would be life-threatening or limb-threatening if present alone; and (iv) an Injury Severity Score (ISS) ≥ 16.
2. Traumatic induced coagulopathy (TIC) was diagnosed according to the Chinese diagnostic criteria for TIC: (i) prothrombin time (PT) > 18 s; (ii) activated partial thromboplastin time (APTT) > 60 s; (iii) thrombin time (TT) > 15 s; or (iv) prothrombin ratio (PTr) > 1.2. TIC was diagnosed if any one of the above criteria was met.

#### 2.1.2 Inclusion criteria

(i) Definite trauma meeting the diagnostic criteria for polytrauma; (ii) Age over 18 years with good physical condition; (iii) time from injury to transfer/admission to our hospital ≤24 h; (iv) good compliance and provision of written informed consent.

#### 2.1.3 Exclusion criteria

(i) Prior history of clearly diagnosed severe disease or major trauma; (ii) self-discharge or treatment withdrawal within 24 h after admission; (iii) pre-existing hematologic disorders or long-term use of anticoagulants/antiplatelet agents; (iv) history of chronic alcohol abuse or malignant tumors; (v) pregnancy; (vi) poor compliance or unwillingness to cooperate with the study.

### 2.2 Variable definitions and sample size considerations

The grouping variable was the occurrence of TIC. Covariates included demographic characteristics (sex and age) and comorbidities (hypertension, diabetes mellitus, and coronary heart disease).

Candidate predictors included: hematological parameters (initial measurements within 24 h of admission): white blood cell count (WBC), hemoglobin (HGB), platelet count (PLT), hematocrit (HCT); liver and renal function indicators: total bilirubin (TBIL), aspartate aminotransferase (AST), alanine aminotransferase (ALT), uric acid (UA), urea (UREA), and creatinine (CREA); coagulation tests: PT, prothrombin ratio (PTR), TT, prothrombin activity (PTA), international normalized ratio (INR), fibrinogen (FIB), and APTT; blood gas indices: pH, lactate (Lac), bicarbonate (HCO−), oxygenation index (P/F), alveolar–arterial oxygen gradient (AaDO2<), and ionized calcium (Ca2+). In addition, the Acute Injury Scale AIS and ISS were calculated for each patient. According to the Events Per Variable (EPV) principle, the final model was constructed such that there were at least 10 outcome events (TIC) per predictor variable.

### 2.3 Statistical analysis

All statistical analyses and figure generation were performed using R software (version 4.4.3). Statistical methods were selected according to variable type and distribution. Categorical variables are presented as frequency and percentage (%) and were compared using the chi-square (*χ*^2^) test or Fisher's exact test. Continuous variables with a normal distribution are expressed as mean ± standard deviation (mean ± SD) and compared using the independent-samples t-test; non-normally distributed continuous variables are reported as median and interquartile range [M (IQR)] and compared using the Mann–Whitney U test.For continuous and categorical variables with missing data, multiple imputation was performed. The primary modeling analyses were then repeated in the imputed datasets to assess the robustness of the results.

To identify predictors, univariate logistic regression analyses were first conducted for all variables. Variables with *P* < 0.05 were entered into a least absolute shrinkage and selection operator (LASSO) regression model to select the most promising predictors while minimizing overfitting. Subsequently, multivariable logistic regression analysis was performed on the selected variables to build the predictive model. Model performance was comprehensively assessed using the area under the receiver operating characteristic curve (AUC) for discrimination, calibration curves for calibration, and decision curve analysis (DCA) for clinical net benefit across different threshold probabilities. All hypothesis tests were two-sided, and *P* < 0.05 was considered statistically significant.

## 3 Results

### 3.1 Baseline characteristics

Among 183 patients with polytrauma, 123 (67.21%) developed trauma-induced coagulopathy (TIC) and 60 (32.79%) did not. Baseline demographics (sex and age) were comparable between groups. On admission, the TIC group presented with significantly lower systolic and diastolic blood pressure than the non-TIC group (both *P* < 0.05), whereas temperature, heart rate, respiratory rate, and peripheral oxygen saturation were similar between groups (all *P* > 0.05). Comorbidities did not differ significantly between groups (all *P* > 0.05).

Laboratory profiling revealed a distinct pattern consistent with coagulopathy and hypoperfusion in the TIC group. Specifically, RBC, platelet count, hematocrit, arterial pH, bicarbonate, prothrombin activity, and fibrinogen levels were significantly lower (all *P* < 0.05). Conversely, WBC, total bilirubin, lactate, and coagulation time-based indices (PT, TT, APTT), as well as INR and prothrombin ratio, were significantly higher in the TIC group (all *P* < 0.05). With respect to injury burden, both AIS and ISS were markedly higher in the TIC group than in the non-TIC group (*P* < 0.001). Clinically, patients with TIC more frequently underwent emergency surgery (31.71% vs. 16.67%, *P* = 0.034) and exhibited a significantly lower survival rate (65.85% vs. 85.00%, *P* = 0.008). Detailed baseline characteristics are summarized in Table 1.

**Table 1:** Baseline characteristics of patients in the study.

Characteristic	Non-Traumatic-coagulopathy *N* = 60	Traumatic-coagulopathy *N* = 123	*p*-value
Sex			0.738
Male	39 (65.00%)	84 (68.29%)	
Female	21 (35.00%)	39 (31.71%)	
Age(years), mean ± SD	50.03 ± 13.59	49.93 ± 15.84	0.965
Temperature( °C), median (Q1, Q3)	36.50 (36.30, 36.60)	36.50 (36.30, 36.70)	0.916
Pulse(bpm), mean ± SD	100.90 ± 22.94	104.93 ± 25.96	0.287
Systolic Pressure(mmHg), mean ± SD	114.18 ± 27.89	104.93 ± 31.37	0.045
Diastolic Pressure(mmHg), median (Q1, Q3)	69.00 (57.00, 84.50)	60.00 (47.00, 76.00)	0.005
Respiratory Rate(bpm), median (Q1, Q3)	20.00 (18.00, 22.00)	20.00 (19.00, 24.00)	0.488
Oxygen Saturation(%), median (Q1, Q3)	94.00 (89.00, 96.00)	93.00 (89.00, 96.00)	0.814
High Blood Pressure			0.819
No	51 (85.00%)	107 (86.99%)	
Yes	9 (15.00%)	16 (13.01%)	
Diabetes Mellitus			0.276
No	59 (98.33%)	116 (94.31%)	
Yes	1 (1.67%)	7 (5.69%)	
Coronary Heart Disease			0.226
No	58 (96.67%)	112 (91.06%)	
Yes	2 (3.33%)	11 (8.94%)	
P/F Ratio, median (Q1, Q3)	270.50 (199.00, 376.85)	268.00 (191.00, 350.00)	0.947
pH, median (Q1, Q3)	7.39 (7.34, 7.43)	7.33 (7.25, 7.38)	<0.001
AaDO2(mmHg), median (Q1, Q3)	81.70 (53.95, 107.10)	94.00 (68.00, 130.00)	0.125
Lactate(mmol/L), median (Q1, Q3)	2.65 (1.55, 4.45)	3.90 (2.40, 6.90)	0.002
Calcium(mmol/L), median (Q1, Q3)	1.15 (1.11, 1.18)	1.14 (1.10, 1.18)	0.483
Bicarbonate(mmol/L), mean ± SD	20.49 ± 4.36	17.46 ± 3.97	<0.001
Total Bilirubin(umol/L), median (Q1, Q3)	12.10 (10.05, 17.70)	11.00 (7.80, 15.60)	0.017
Aspartate Transaminase(U/L), median (Q1, Q3)	54.85 (29.05, 104.40)	74.60 (43.30, 134.50)	0.056
Alanine Transaminase(U/L), median (Q1, Q3)	31.85 (23.30, 60.10)	39.90 (26.20, 69.80)	0.304
Uric Acid(umol/L), median (Q1, Q3)	325.00 (241.50, 413.50)	347.00 (278.00, 417.00)	0.299
Urea(mmol/L), median (Q1, Q3)	5.82 (4.86, 7.72)	5.96 (4.89, 7.17)	0.591
Creatinine(umol/L), median (Q1, Q3)	67.40 (55.70, 89.25)	73.60 (62.20, 97.30)	0.054
White Blood Cells(*109/L), median (Q1, Q3)	13.79 (10.88, 19.10)	17.03 (12.97, 21.67)	0.013
Red Blood Cells(*1012/L), median (Q1, Q3)	3.60 (3.26, 4.28)	3.06 (2.40, 3.70)	<0.001
Platelet Count(*109/L), median (Q1, Q3)	206.00 (146.00, 259.50)	156.00 (112.00, 211.00)	<0.001
Hematocrit(%), median (Q1, Q3)	32.65 (28.90, 38.25)	27.60 (22.50, 34.50)	<0.001
Prothrombin Time(s), median (Q1, Q3)	12.20 (11.45, 12.80)	14.60 (13.30, 16.70)	<0.001
Prothrombin Time Ratio, median (Q1, Q3)	1.00 (1.00, 1.10)	1.30 (1.20, 1.50)	<0.001
Thrombin Time(s), median (Q1, Q3)	14.00 (13.00, 14.00)	18.00 (17.00, 20.00)	<0.001
Prothrombin Activity(%), mean ± SD	86.50 ± 12.15	64.15 ± 16.63	<0.001
International Normalized Ratio, median (Q1, Q3)	1.11 (1.05, 1.17)	1.33 (1.21, 1.52)	<0.001
Fibrinogen(g/L), median (Q1, Q3)	2.31 (1.85, 2.95)	1.34 (0.98, 1.66)	<0.001
Activated Partial Thromboplastin Time(s), median (Q1, Q3)	27.90 (25.55, 29.30)	30.70 (27.80, 35.40)	<0.001
Abbreviated Injury Scale (AIS), median (Q1, Q3)	7.00 (7.00, 9.00)	10.00 (8.00, 12.00)	<0.001
Injury Severity Score(ISS), median (Q1, Q3)	19.00 (18.00, 26.00)	32.00 (26.00, 41.00)	<0.001
Emergency Surgery			0.034
No	50 (83.33%)	84 (68.29%)	
Yes	10 (16.67%)	39 (31.71%)	
Survival			0.008
No	9 (15.00%)	42 (34.15%)	
Yes	51 (85.00%)	81 (65.85%)	

### 3.2 Logistic regression analysis

To identify independent factors associated with TIC, we performed univariable and multivariable logistic regression analyses. In univariable analyses, multiple variables were significantly associated with TIC, including diastolic blood pressure, arterial pH, bicarbonate, lactate (Lac), total bilirubin (TBIL), white blood cell count (WBC), red blood cell count (RBC), platelet count (PLT), hematocrit (HCT), coagulation-related parameters (PT, TT, prothrombin activity [PTA], fibrinogen [FIB], and APTT), as well as injury severity indices (AIS and ISS) and emergency surgery (all *P* < 0.05) (Table 2).

**Table 2:** Logistic regression analysis for TIC.

Characteristic	Univariable		Multivariable	
	OR (95% CI)	P值	OR (95% CI)	P值
Sex	0.862 (0.451–1.670)	0.656		
Age	1.000 (0.979–1.020)	0.967		
T	0.933 (0.451–2.031)	0.851		
P	1.007 (0.994–1.019)	0.306		
Systolic pressure	0.990 (0.979–1.000)	0.056		
Diastolic pressure	0.985 (0.971–0.999)	0.032	0.980 (0.960∼1.000)	0.081
R	1.001 (0.952–1.055)	0.982		
SPO2	0.991 (0.957–1.021)	0.589		
High blood pressure	0.847 (0.357–2.123)	0.713		
Diabetes mellitus	3.560 (0.613–67.360)	0.240		
Coronary heart disease	2.848 (0.733–18.803)	0.183		
P/F	0.999 (0.996–1.002)	0.476		
PH	0.000 (0.000–0.001)	<0.001	4.034（2.059–7.901）	<0.001
AaDO2	1.003 (0.998–1.007)	0.265		
lac	1.169 (1.042–1.330)	0.012		
Ca2+	1.018 (0.976–NA)	0.622		
HCO3-	0.830 (0.756–0.903)	<0.001		
TBIL	0.958 (0.924–0.988)	0.013	0.971 (0.940∼1.013)	0.185
AST	1.001 (0.999–1.003)	0.314		
ALT	1.001 (0.999–1.003)	0.566		
UA	1.001 (0.999–1.004)	0.338		
UREA	0.950 (0.833–1.080)	0.421		
CREA	1.008 (0.998–1.020)	0.167		
WBC	1.059 (1.008–1.117)	0.026		
RBC	0.388 (0.246–0.587)	<0.001	0.450 (0.251∼0.811)	0.008
PLT	0.992 (0.987–0.996)	<0.001		
HCT	0.935 (0.895–0.973)	0.002		
PT	3.023 (2.168–4.527)	<0.001		
TT	4.352 (2.869–7.502)	<0.001		
PTA	0.889 (0.853–0.920)	<0.001		
FIB	0.701 (0.519–0.916)	0.015		
APTT	1.055 (1.016–1.103)	0.011		
AIS	1.489 (1.279–1.762)	<0.001		
ISS	1.190 (1.127–1.268)	<0.001	1.232 (1.138∼1.327)	<0.001
Emergency surgery	2.321 (1.100–5.284)	0.034	3.750 (1.311∼10.752)	0.014

T, Temperature; P, Pulse; R, Respiratory Rate; P/F, P/F Ratio; lac, Lactate; Ca2+, Calcium; HCO3-, Bicarbonate; TBIL, Total Bilirubin; AST, Aspartate Transaminase; ALT, Alanine Transaminase; UA, Uric Acid; UREA, Urea; CREA, Creatinine; WBC, White Blood Cells; RBC, Red Blood Cells; PLT, Platelet Count; HCT, Hematocrit; PT, Prothrombin Time; PTA, Prothrombin Time Ratio; TT, Thrombin Time; FIB, Fibrinogen; APTT, Activated Partial Thromboplastin Time; AIS, Abbreviated Injury Scale (AIS); ISS, Injury Severity Score.

**Figure 1:**
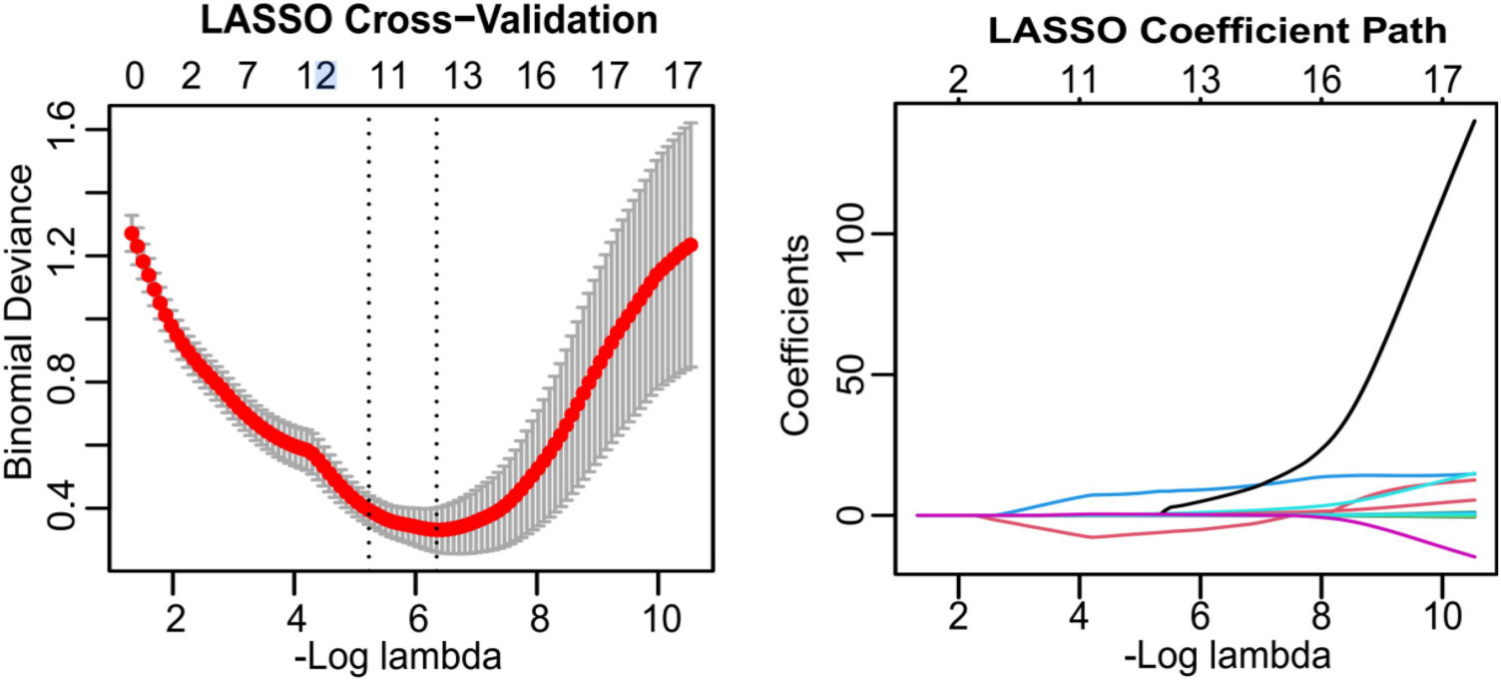
LASSO regression With cross-validation for optimal *λ* selection.

Subsequently, variables with statistical significance in univariable analyses were entered into a LASSO regression model, with the optimal penalty parameter determined at *λ* = 13 (Figure 1). To mitigate multicollinearity, variance inflation factors (VIFs) were calculated for LASSO-selected predictors; variables with VIF >5 were excluded prior to multivariable modeling.

In the final multivariable logistic regression model, emergency surgery, arterial pH, RBC, and ISS remained independently associated with TIC (Table 2). Specifically, emergency surgery was associated with increased odds of TIC (OR = 3.75, 95% CI: 1.31–10.75; *P* = 0.014), whereas higher arterial pH (OR < 1, *P* < 0.001) and higher RBC (OR = 0.45, 95% CI: 0.25–0.81; *P* = 0.008) were protective. ISS was positively associated with TIC risk (OR = 1.23, 95% CI: 1.14–1.33; *P* < 0.001). Notably, although diastolic blood pressure and TBIL were significant in univariable analyses, neither remained statistically significant after multivariable adjustment (both *P* > 0.05) and were not included in the final model to maintain parsimony.

### 3.3 Development and validation of the prediction model

Based on the independent predictors identified by multivariable logistic regression, we developed a prediction model to estimate the risk of TIC. The model's predicted probability (P) was calculated using the logistic regression equation: Logit(P) = 86.20 + 1.32×(Emergency surgery)−11.79×pH−0.79×RBC + 0.21×ISS.

**Figure 2:**
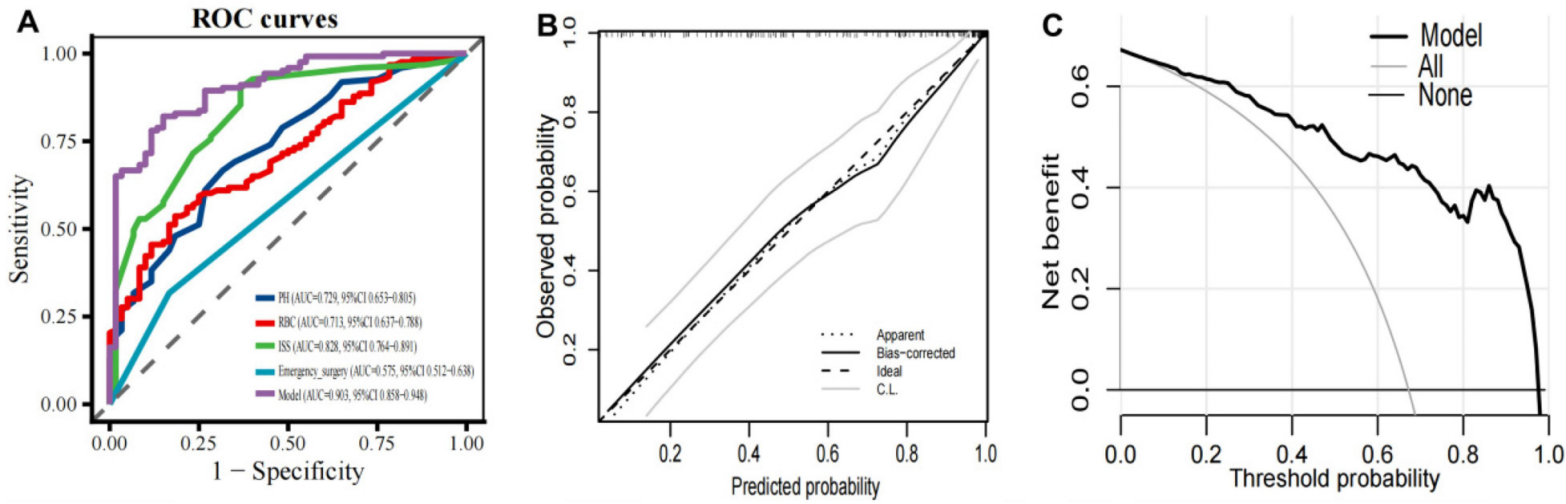
Development and performance evaluation of the prediction model. The predicted probability **(P)** was calculated using the following logistic regression equation:X = 86.20 + 1.32×Emergency surgery−11.79×pH−0.79×RBC + 0.21×ISS, *P* = 1/[1 + exp(−X)]. where P represents the predicted probability of TIC occurrence. Emergency surgery was entered as a binary variable, whereas pH, RBC, and ISS were entered as their original continuous values. **(A)** ROC curve analysis of the model and individual variables; **(B)** Calibration curve analysis of the model; **(C)** Decision curve analysis of the model.

To evaluate its discriminative performance, we generated the receiver operating characteristic (ROC) curve (Figure 2A). The model demonstrated excellent discrimination, with an area under the curve (AUC) of 0.903 (95% CI: 0.858–0.948). The optimal cut-off value determined by the Youden index was 0.651, yielding a sensitivity of 81.3% and a specificity of 86.7%. Furthermore, the model demonstrated superior performance compared with each individual predictor used alone, indicating strong discriminatory and classification capability. Apparent calibration analysis showed a calibration intercept of 0.000 and a calibration slope of 1.000. The Brier score was 0.116, suggesting acceptable overall predictive accuracy.

Model accuracy and potential clinical utility were further assessed using calibration analysis and decision curve analysis (DCA). The calibration curve showed close agreement between the predicted and observed probabilities across the risk spectrum, with the curve approaching the ideal 45-degree line, suggesting good calibration (Figure 2B). In addition, DCA indicated that the model yielded a higher net benefit across a broad range of threshold probabilities, supporting its potential value in facilitating clinical decision-making (Figure 2C).

## 4 Discussion

In this prospective cohort study of adult polytrauma patients older than 18 years,we developed and validated a parsimonious prediction model incorporating four parameters—emergency surgery, arterial pH, red blood cell count (RBC), and Injury Severity Score (ISS)—to individually estimate the risk of traumatic-induced coagulopathy (TIC) among patients with polytrauma. The model demonstrated robust discrimination, good calibration, and clinically meaningful utility, indicating its potential to support early risk stratification and targeted intervention.

Compared with previous TIC prediction studies (13–16), most of which were retrospective and used variable predictor sets, our prospective design and inclusion of a broader range of routinely available clinical parameters strengthen the evidence base. For example, in a large retrospective multicenter analysis of the PROMMTT dataset, Cohen et al. identified an ISS >15 in combination with a base deficit <-6 as a key driver of acute traumatic coagulopathy; however, emergency surgery and red blood cell count were not examined as independent predictors in that study (17). Our prospective model extends these earlier findings by incorporating emergency surgery and RBC count as additional predictors, which may further enhance predictive accuracy.

The final model included four key predictors: emergency surgery, arterial pH, RBC, and ISS. Emergency surgery was independently associated with a substantially increased risk of TIC. It is important to consider whether emergency surgery acts primarily as a marker of injury severity (i.e., reflecting the need for operative control of severe hemorrhage) or as a direct causal contributor to TIC. In our view, both interpretations are relevant: the underlying injuries that mandate emergency surgery (e.g., major vascular or solid organ disruption) are themselves potent triggers of coagulopathy, but the surgical procedure—particularly when performed before coagulopathy is corrected—may impose an additional “second hit” through surgical stress, anesthesia, hypothermia, and blood loss, thereby exacerbating existing coagulopathy (18, 19). These findings underscore the importance of rapid, dynamic coagulation monitoring and goal-directed resuscitation in the perioperative management of polytrauma patients requiring emergency surgery.

ISS emerged as one of the strongest predictors of TIC, consistent with the established concept of trauma mortality and with prior evidence (20, 21). ISS reflects the overall burden and extent of tissue injury. Extensive injury may simultaneously promote TIC through multiple mechanisms, including tissue factor release with activation of the extrinsic coagulation pathway, hyperfibrinolysis, and endothelial glycocalyx shedding, collectively driving the initiation and progression of TIC (22). Accordingly, patients with high ISS should be considered at particularly high risk and should undergo prompt screening and close monitoring for TIC. Notably, arterial pH was a highly sensitive predictor of TIC in our model. Severe acidosis (low pH) can impair hemostasis via several pathways, including suppression of enzymatic activity of coagulation factors (notably FVIIa and FVa), reduced platelet aggregation, and decreased fibrinogen stability. Our clinical findings corroborate experimental evidence that acidosis is a central contributor to the development and deterioration of TIC (23, 24). In clinical practice, continuous monitoring and timely correction of acidosis should be viewed not only as a fundamental resuscitation target but also as a key component in the prevention and management of TIC. Higher RBC levels showed a protective association. Traditionally, the contribution of RBCs to hemostasis has been underestimated. Emerging evidence suggests that RBCs may promote hemostasis through both rheological mechanisms (e.g., margination of platelets toward the vessel wall) and biochemical pathways (e.g., ADP release), thereby facilitating platelet recruitment and clot formation (25–27). Our results provide clinical support for these mechanisms and imply that trauma resuscitation strategies should avoid unnecessary or excessive crystalloid infusion that may induce hemodilution. When clinically indicated, timely RBC transfusion may help preserve hemostatic function.

Although total bilirubin was significantly associated with TIC in univariate analysis, it did not retain significance in the multivariate model. This finding suggests that bilirubin may be confounded by other variables more directly linked to TIC pathophysiology, such as tissue injury severity (reflected by ISS) or shock markers (e.g., pH, lactate). Elevated bilirubin in polytrauma patients can arise from multiple sources, including transfusion-related hemolysis, hepatic hypoperfusion, or pre-existing hepatobiliary conditions, and its effect on coagulation may be mediated through these pathways. The loss of significance after adjustment indicates that bilirubin is not an independent predictor of TIC in our cohort, consistent with the view that it serves primarily as a marker of underlying injury burden rather than a direct driver of coagulopathy.

Several limitations should be considered. This was a single-center retrospective study with a modest sample size, and the patient population may not be fully representative of other settings, introducing potential selection bias (including age restriction to >18 years). The absence of prehospital fluid or transfusion data, the use of only conventional coagulation assays, and the lack of external validation are additional limitations. Moreover, there remains the possibility of overfitting, as model development and testing were performed in the same cohort. Given that this is a single-center model development study, some of our claims—particularly those regarding clinical implementation—should be viewed as preliminary. In particular, the suggestion that the model could be used in resource-limited settings, while interesting, remains speculative without external validation. Finally, although the model's performance was acceptable, unidentified predictors (e.g., procalcitonin) may exist, and the model must be externally validated in independent prospective cohorts across diverse geographic and healthcare environments before clinical implementation.In light of these limitations, future work will focus on several directions. First, we plan to establish multicenter collaborations to conduct large-scale external validation, which is essential for clinical translation. Second, we aim to implement the model as a user-friendly web-based calculator or mobile application to facilitate rapid deployment in emergency settings, particularly in primary care and resource-limited institutions. although such efforts will be contingent on successful external validation.Ultimately, these efforts may enable earlier, more precise prevention and treatment of TIC and reduce the public health burden of this lethal complication in regions where trauma is highly prevalent.

In summary, For adult patients with severe multiple trauma，high ISS, the need for emergency surgery, severe acidosis, and low RBC levels are key warning signals for the development of TIC. We successfully developed a functional prediction model that enables accurate, individualized quantification of TIC risk. The model demonstrated good discrimination and calibration and provided meaningful net benefit in decision curve analysis.However, given the limitations noted above, external validation in independent cohorts is required before any clinical application. If further validated in future prospective studies, it may become a valuable tool to optimize allocation of medical resources and facilitate early diagnosis and timely intervention for TIC, thereby reducing the burden of this disabling complication in trauma-endemic regions.

## 5 Conclusion

In conclusion, this prospective cohort study identified emergency surgery, pH, RBC, and ISS as predictors of TIC in polytrauma patients aged >18 years. The derived model demonstrated promising discrimination and calibration, but external validation is required before clinical implementation.

## Data Availability

The original contributions presented in the study are included in the article/Supplementary Material, further inquiries can be directed to the corresponding author.
